# Synthesis of Composite Titanate Photocatalyst via Molten Salt Processing and Its Enhanced Photocatalytic Properties

**DOI:** 10.3390/nano13222944

**Published:** 2023-11-14

**Authors:** Yan Cheng, Chenxi Li, Shindume Lomboleni Hamukwaya, Guangdong Huang, Zengying Zhao

**Affiliations:** 1 School of Science, China University of Geosciences (Beijing), Beijing 100083, China; 2 School of Engineering and the Built Environment, University of Namibia, Ongwediva, Windhoek 33004, Namibia

**Keywords:** titanate, photocatalysis, molten salt, methylene blue, nitrate

## Abstract

Photocatalysis plays a pivotal role in environmental remediation and energy production and improving the efficiency of photocatalysts, yet enhancing its efficiency remains a challenge. Titanate has been claimed to be a very promising material amongst various photocatalysts in recent years. In this work, a novel composite photocatalyst of sodium titanate and potassium titanate was synthesized via a simple hydrothermal and molten salt calcination method. Low melting point nitrate was added in the calcination process, which helps reduce the calcination temperature. The as-prepared composite sample showed excellent photocatalytic performance compared with commercial P25 in the visible light range. According to the characterization of XRD, SEM, TEM, BET, UV–Vis, and photocatalytic property testing, the composite’s photocatalytic performance results are due to the dual optimization brought about by the layered structure and composite of titanium salts forming a heterojunction. We believe that the composite has significant application potential for the use of titanate in the field of photocatalysis. Notably, this study employed well-documented synthesis methods and adhered to established protocols for experimental procedures.

## 1. Introduction

With industrial progress, the discharge of organic pollutants has caused serious environmental pollution, posing a potential threat to ecosystems and human health. Methylene blue (MB) is an organic dye widely used in the textile, dye, and pharmaceutical industries. It has strong light absorption and is difficult to degrade. With the progress of industry, the emission of methylene blue has caused serious environmental pollution, posing a potential threat to ecosystems and human health [1,2]. Solar energy, as an endless clean energy source, has always been an important topic of concern in academia. In 1972, Fujishima and Honda accidentally discovered that titanium dioxide can decompose water into H_2_ and O_2_ under ultraviolet light, marking the birth of photocatalysis technology [3]. Photocatalysts are photosensitive materials that can generate photoelectrons and reactive oxygen species under visible or ultraviolet light irradiation, thereby degrading organic pollutants. Photocatalysts have the advantages of high efficiency, no secondary pollution, and recyclability, so they are considered to be an environmentally friendly technology with important application potential. Since the discovery of the enormous potential of photocatalysts, photocatalysis technology has gradually been applied to the degradation of pollutants and CO_2_ reduction, achieving significant results [4,5,6]. Photocatalysis technology is currently regarded as one of the most effective means of addressing energy and environmental pollution issues.

The key to the application of photocatalysis is to find efficient semiconductor materials. These materials need to have a suitable bandgap width, strong light absorption capacity, and good stability. With the advancement of this field, people have developed different types of materials, such as sulfides [7,8], nitrides [9,10], and oxides [11,12], and have conducted in-depth research and application studies on their photocatalytic performance. Titanium dioxide (TiO_2_) has attracted extensive research interest in the fields of energy and environment due to its chemical stability, non-toxicity, low cost, wide availability, and excellent photoelectric properties. However, the main limiting factors of its photocatalytic efficacy are the relatively high charge carrier recombination rate and the broad bandgap (3–3.2 eV), which both restrict its light absorption [13]. As a new prospect in the field of photocatalysis, metal-modified titanates have demonstrated optimistic performance in degrading organic pollutants [14]. It is known that layered semiconductor compounds have a special interlayer two-dimensional nanostructure that can serve as a suitable reaction site, effectively suppressing the reverse reaction of photogenerated electron and hole recombination, thereby improving photocatalytic activity. Layered titanium oxides are an excellent representative among many TiO_2_-based semiconductor photocatalysts. These oxides are composed of octahedral TiO_6_ connected in a co-edge or co-angle manner to form a two-dimensional layered oxide with a negative charge and contain alkali metal cations between layers. It can be represented by the following formula A_2_Ti_n_O_2n+1_, where 1 < n < 9, and A represents H, Li, Na, K, and Cs [15,16,17,18]. Layered titanium oxides are widely studied due to their high activity and good anti-photo-corrosion properties. They have extremely high ion exchange, transfer, and adsorption capabilities. Their unique interlayer two-dimensional nanostructure can be used as a suitable reaction site to suppress photogenerated electron–hole recombination effectively and improve catalytic reaction efficiency [19,20,21]. As a type of titanium salt, potassium titanate plays an important role in various fields due to its unique crystal structure, which can take the form of layered, fibrous, or whisker-like shapes [22]. The chemical molecular formula of potassium titanate crystal is K_2_O·nTiO_2_, where n = 1, 2, 4, 6, 8. Different values of n result in significant structural differences. Generally, when n = 2, 4, the potassium titanate crystal has a layered structure, which is the structure we hope to obtain. Moreover, due to its excellent properties, such as high photocatalytic activity, potassium titanate is widely used in industry. Takaya et al. studied the nanoneedle-shaped potassium titanate crystal, which has high photocatalytic activity and is approximately three times more effective in photocatalytic degradation of methylene blue than TiO_2_ film within 20 h [23]. Gao et al. also obtained potassium tetra-titanate (K_2_Ti_4_O_9_) through a high-temperature solid-phase reaction and doped K_2_Ti_4_O_9_ with nitrogen using urea as a nitrogen source. The results showed that the catalyst’s absorption capacity for visible light was improved after nitrogen doping, and the photocatalytic performance made significant progress [24]. Cui et al. synthesized Cd_1−x_Zn_x_S/K_2_Ti_4_O_9_ via the deposition–precipitation method, where Cd_0.8_Zn_0.2_S had the highest photocatalytic RhB activity, with a degradation rate of 95% [25]. This paragraph describes the research on sodium metatitanate (Na_2_TiO_3_). Compared with other titanium sodiums, there is less research on Na_2_TiO_3_, but its layered structure is very attractive and stable [26,27]. Kobayashi et al. studied the Na_2_TiO_3_-NaMnO_2_ binary system and found that the reversibility of anion redox was significantly improved after multiple experiments. Na_1.14_Mn_0.57_Ti_0.29_O_2_ in this binary system provides a large reversible capacity of about 200 mA h g^−1^ [28]. In 2023, Cao et al. constructed a rich Na-layered oxide Na_2_TiO_3_ as a multifunctional coating on the surface of Na_0.44_MnO_2_ nanorods. Na_2_TiO_3_ can serve not only as a reservoir for Na^+^ but also as a protective layer to prevent Na_0.44_MnO_2_ from being corroded by the electrolyte. In addition, the Ti-doped Na_0.44_MnO_2_ transition layer provides an additional Na^+^ diffusion path along the radial direction of the nanorod [29]. It can be seen that titanium oxides have great potential in the field of photocatalysis. Currently, the main methods include the melting method [30], the sintering method [31], the Kneading Drying Calcination (KDC) method [15], and the hydrothermal method [32]^.^ However, the problem with its application is that its preparation often requires high-temperature conditions. Therefore, it becomes necessary to reduce the calcination temperature of layered titanium oxide.

Photocatalysis is an emerging technology with the potential to address various environmental and energy-related challenges. Titanate-based photocatalysts have shown promise due to their excellent photocatalytic activity. However, enhancing their performance remains a crucial research goal. Molten salt processing offers a unique method for synthesizing composite photocatalysts that can potentially overcome the limitations of single-phase materials. This study explores the application of molten salt processing to create a composite titanate photocatalyst and investigates its photocatalytic properties.

This study used a novel synthetic salt melting technique to replace carbonates with low melting point nitrates to lower the calcination temperature. In addition, previous studies have investigated the application of TiO_2_ in photocatalysis, such as degradation of pollutants and hydrogen production [20,33,34,35,36]. There are few reports about the photocatalysis of titanate. The molten salt method used in this paper reduces the calcination temperature and reaction difficulty, paving the way for the use of titanium salts in photocatalytic applications.

## 2. Materials and Methods

### 2.1. Materials

Titanium tetrabutoxide ([CH_3_(CH_2_)_3_O]_4_Ti, chemically pure) was purchased from Beijing Yili Fine Chemicals Co., Ltd. (Beijing, China) Acetic acid (CH_3_COOH, analytical pure) and sodium nitrate (NaNO_3_, analytical pure) were obtained from Beijing Chemical Plant (Beijing, China). Urea (H_2_NCONH_2_, analytical pure) was purchased from Xilong Chemical Co., Ltd. (Shanghai, China), and potassium nitrate (KNO_3_, analytical pure) was obtained from Modern Oriental Technology Development Co., Ltd. (Beijing, China). Finally, TiO_2_ (P25) was used as a comparison for the photocatalytic performance test of the titanate samples. All materials were utilized without additional purification.

### 2.2. Preparation of Titanate

An amount of 50 mL of acetic acid solution was added to a PTFE reactor, and 20 mL of tetrabutyl titanate solution was slowly added with uniform stirring. Then, a urea solution (0.04 mol) was added and stirred for 30 min. The combination was treated to a 24 h hydrothermal reaction at a temperature of 150 °C. After centrifugation and drying, the sample was mixed with NaNO_3_-KNO_3_ mixed salt in a molar ratio of 1:5, where the molar ratio of NaNO_3_ to KNO_3_ was 1:1. Based on the previous exploration of our research group, it is difficult to generate titanate products at 600 °C [37,38,39]. Therefore, the corresponding titanium salt samples were obtained via calcining at 600, 650, 700, 750, and 800 °C for 4 h after sufficient washing and drying.

### 2.3. Characterization

The phase and crystal structure of the powder were analyzed via X-ray diffraction (XRD) using a Rigaku D/max instrument. Cu Kα radiation (λ = 0.154 nm) was employed, and the scanning rate was set at 4°/min. Sample morphology was characterized using scanning electron microscopy (SEM) and transmission electron microscopy (TEM). The material’s specific surface area (BET) was determined by evaluating the N_2_ adsorption isotherm using the BET method. Pore distribution and volume were derived from the desorption isotherm data.

The sample visible light absorption performance was obtained via a scanning test using a UV-visible spectrophotometer (Perkin Elmer, Lambda 900), with a scanning range of 200–800 nm and barium sulfate (BaSO_4_) as the background. The bandgap width can be calculated by extending the linear component of the 1/2 Ahv against the hv plot to the energy axis focus.

An amount of 50 mg of catalyst was introduced into 50 mL of 5 mg/L MB solution to examine the photocatalytic performance of the sample degradation effect under visible light using TiO_2_ (P25) as a reference. The sample photocatalytic performance was specifically examined via degradation of 5 mg/L MB under visible light illumination. A 500 W xenon lamp was utilized as the light source above the photochemical reaction apparatus, and a 420 nm filter was added to provide visible light. Before the light reaction, the MB solution with catalyst addition was ultrasonically agitated in the dark for 30 min to produce adsorption–desorption equilibrium across the photocatalyst and MB. After the dark reaction, the sample was placed on a multi-tube stirrer for photocatalytic degradation under visible light irradiation. The solution was centrifuged every 30 min to remove the catalyst, and the upper clear liquid was tested for absorbance at 662 nm wavelength. The instruments used in the above operation process are a UV-visible spectrophotometer (772s, Shanghai Instrument Ltd., Shanghai, China) and a photochemical reactor XPA-7 (Xu Jiang Instrument Ltd., Nanjing, China).

## 3. Results and Discussion

### 3.1. Structure and Morphology

**Figure 1:**
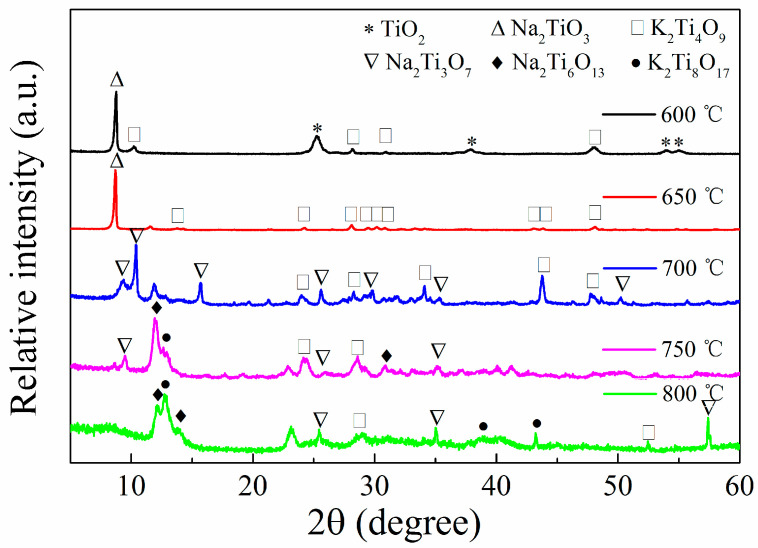
XRD patterns of titanate samples calcined at different temperatures.

In accordance with the experimental results, we prepared titanium salt samples at different calcination temperatures and analyzed them via XRD. As shown in Figure 1, the 600 °C sample still contains rutile phase titanium dioxide (2θ = 25.30°, 37.87°, JCPDS. No 21-1272) and produces sodium metatitanate and potassium metatitanate (JCPDS. No 11-0291 and 13-0447); as the calcination temperature increases, the titanium dioxide phase disappears, and the variety of substances in the sample increases. When the calcination temperature reaches 650 °C, the titanium dioxide phase disappears, and the sample mainly contains sodium metatitanate and potassium metatitanate; when the temperature reaches 700 °C, the sample mainly contains sodium trititanate (JCPDS. No 14-0085) and potassium metatitanate; and when the temperature reaches 750 °C, sodium hexatitanate and potassium octatitanate (JCPDS. No 13-0589 and 35-0089) are produced.

According to the XRD pattern, we speculated on the reaction mechanism of titanium salts. At 400–500 °C, nitrate decomposes to produce potassium oxide and sodium oxide; when the temperature reaches 600 °C, K_2_O and Na_2_O react with TiO_2_ to generate potassium metatitanate and sodium metatitanate, and a certain amount of titanium dioxide remains; when the temperature reaches 650 °C, the reaction completely consumes titanium dioxide, and only sodium metatitanate and potassium metatitanate are contained in the sample; as the temperature rises, sodium trititanate may be transformed from sodium metatitanate at 700 °C or directly obtained via reaction between sodium oxide and titanium dioxide; and as the temperature continues to rise, sodium hexatitanate and potassium octatitanate are produced in the sample at 750 °C.

**Figure 2:**
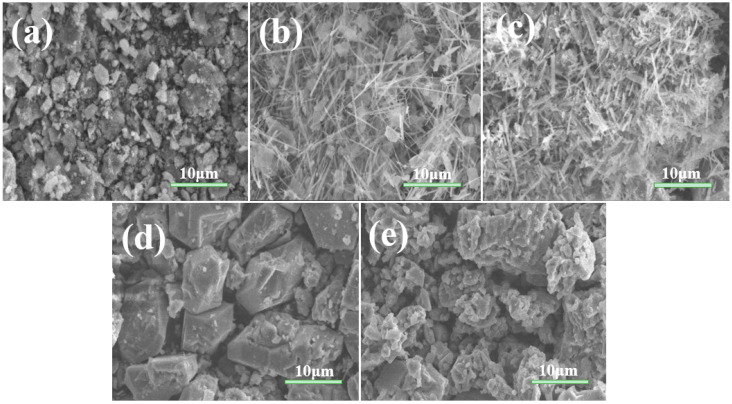
Typical SEM images of titanate samples calcined at various temperatures: (**a**) 600; (**b**) 650; (**c**) 700; (**d**) 750; and (**e**) 800 °C.

As shown in Figure 2, we conducted SEM tests on the titanate samples prepared at different calcination temperatures, which revealed a unique morphology with increased surface area. At 600 °C, we observed that the sample was composed of a large number of particles. These particles were uneven in size and shape, presenting an irregular blocky appearance. In addition, many irregular small particles were adhering to these blocky particles, with sizes ranging from tens to several nanometers. When the temperature rose to 650 °C, a large number of nanobelts were produced. These nanobelts were relatively uniform in morphology but disordered in arrangement. Their surfaces were smooth, edges clear, lengths between 6–13 μm, and widths around 200 nanometers. Notably, there were also some irregular small particles adhering to the nanobelts. However, when the temperature was increased further to 700 °C, the sample only contained nanobelts, and the surface smoothness decreased. By comparing the high-magnification SEM photos of the samples at 650 °C and 700 °C, we found that as the temperature increased, the nanobelts became thicker. When the temperature reached 750 °C, there were no nanobelts in the sample, only large particles with diameters between 5–12 μm. This might be due to a significant reduction in the content of layered potassium titanate and sodium titanate, which hindered the generation of nanobelts. Finally, under 800 °C, we observed small particles adhering to the large particles. This might be due to reaching the melting point of potassium oxide. The reduced fluidity and diffusivity of the molten system led to a relatively slow nucleation and growth rate, resulting in small particles.

**Figure 3:**
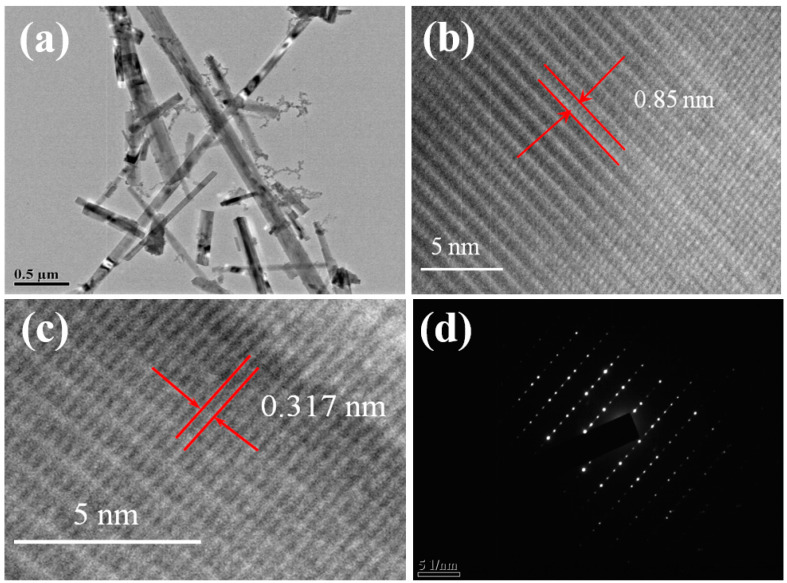
TEM (**a**), HRTEM (**b**,**c**), and SAED (**d**) images of titanate sample calcined at 650 °C.

In order to further observe the morphology of the titanate sample prepared at 650 °C, we conducted a TEM analysis. Figure 3a shows that the titanate is nanobelt-shaped, with some nanocrystals of about 20 nm in diameter attached to the surface. The combination of these two forms of semiconductors can effectively separate photogenerated carriers to different material surfaces. The potassium titanate nanobelt has good conductivity, which can not only effectively collect generated electrons but also provide a more direct conduction path for electrons, thereby improving the photocatalytic efficiency of the sample. Figure 3b shows the HRTEM photo of the sample. From the figure, we can clearly see the layered structure of the sample. This is consistent with the well-known morphology of potassium titanate [40,41,42]. Through calculation, the layer spacing is 0.85 nm, which is much larger than the hydration radius of K^+^ (0.133 nm) [43,44]. This interlayer distance can serve as a channel for K^+^ to enter and exit freely. The dissolution of K^+^ can promote the formation of hydrated tetratitanic acid (H_2_Ti_4_O_9_·12H_2_O) fibers, and this kind of tetratitanic acid fiber has good ion adsorption. From Figure 3c, we can measure that the crystal fringe spacing of the nanobelt is 0.317 nanometers, which completely matches the potassium tetratitanate (211) plane and is consistent with XRD test results. Finally, Figure 3d shows the diffraction pattern of this sample. The point-like diffraction proves the prepared titanate nanobelt is a single-crystal structure.

**Figure 4:**
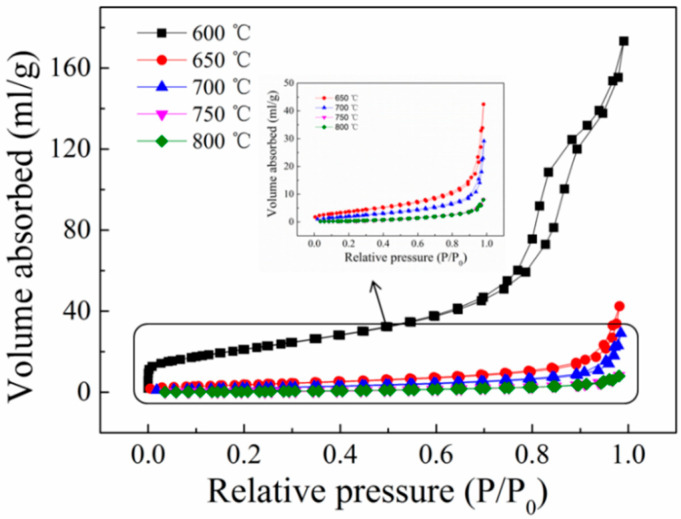
N_2_ adsorption–desorption isotherms of titanate samples calcined at different temperatures.

As one of the basic properties of photocatalysts, its structure can affect photocatalytic performance. The pore structure of the sample can be deduced from the N_2_ adsorption–desorption curve, as illustrated in Figure 4. The sample prepared at a calcination temperature of 600 °C belongs to the type IV adsorption isotherm with an H2-type hysteresis loop, suggesting the existence of mesopores in the sample, as shown in Figure 4. However, the samples prepared at 650 °C, 700 °C, 750 °C, and 800 °C belong to the type II adsorption isotherm, indicating that the sample is non-porous or has a macroporous structure. Notably, the sample prepared at 650 °C has an H3-type hysteresis loop, demonstrating the existence of a sample layered structure [45]. As a result, the synthesized composite titanate photocatalyst exhibited a unique nanostructure with well-dispersed titanate nanoparticles within the salt matrix, as confirmed via XRD, SEM, and TEM analyses.

The specific surface area, pore volume, and pore size of the titanate samples prepared at various calcination temperatures are shown in Table 1. By contrasting the specific surface areas of various samples, we found that the specific surface area of the sample gradually reduces as the calcination temperature increases. There are two reasons for this effect: Firstly, from the morphological point of view (SEM of Figure 2), the sample particles changed from scattered blocks to a mixture of nanoribbons and large particles and then to pure large particles, with the volume increasing and the agglomeration phenomenon obvious. Secondly, from the pore structure point of view (Figure 4), the sample pores changed from mesopores to macropores or no pores, the pore volume decreased, and the sample density increased. Both of them showed a regular trend with the temperature change.

### 3.2. Photocatalytic Performance Test for MB Degradation

**Figure 5:**
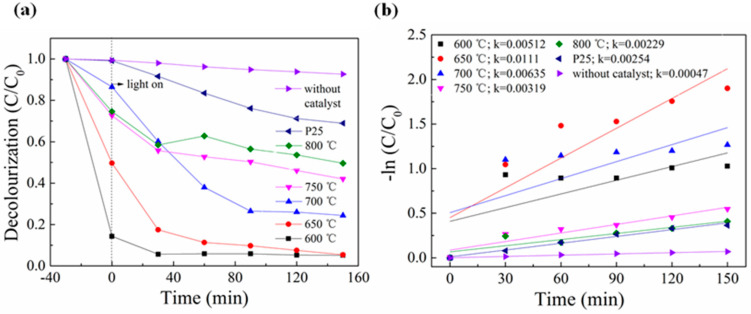
(**a**) MB degradation curves for titanate samples calcined at different temperatures under visible light irradiation and (**b**) their corresponding plots of −ln (C/C_0_).

In this experiment, different titanium salt samples and P25 were prepared at different calcination temperatures to evaluate their photocatalytic activity for the degradation of the organic dye MB under visible light, as shown in Figure 5a. A blank sample was used as a comparison. Figure 5 shows that the degradation rate of the sample without a catalyst was only 8% within 150 min, while the degradation efficiency with a catalyst was significantly improved. With the increased calcination temperature, the titanium salt samples prepared exhibited a pattern of increasing and decreasing MB degradation. Among them, the titanium salt sample prepared at 650 °C had the highest decolorization rate for MB, reducing 95% of MB in solution in 150 min, while P25 had a degradation rate of only 31% for MB solution. In addition, it can be observed from Figure 5 that the adsorption effect of the sample prepared at 600 °C is particularly good, adsorbing 86% of MB in 30 min. Figure 5b shows the linear relationship between reaction time and −ln (C/C_0_), indicating that MB degradation follows first-order reaction kinetics. The rate constants of titanium salt samples and P25 prepared at different calcination temperatures were 0.00512, 0.0111, 0.00635, 0.00319, 0.00229, and 0.00047 min^−1^, respectively. The rate constant of titanium salt samples increased and then decreased with increasing calcination temperature, with the sample prepared at 650 °C exhibiting the highest rate constant, demonstrating that it has the best photocatalytic degradation effect. This improvement is attributed to the increased surface area and reduced charge recombination resulting from the composite structure.

### 3.3. Optical and Electrical Properties

**Figure 6:**
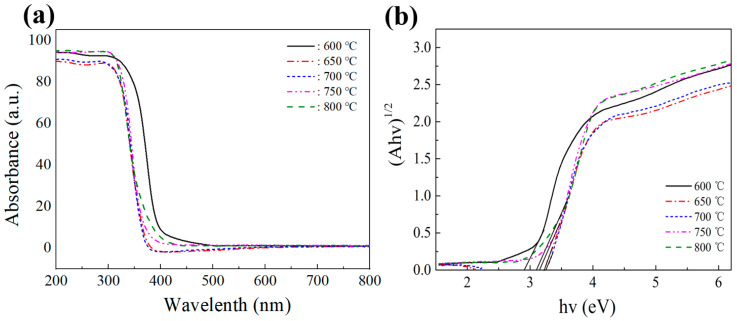
(**a**) UV–Vis absorption spectra of titanate samples calcined at different temperatures, and (**b**) their corresponding Kubelka–Munk curves.

Figure 6a displays the UV-visible absorption spectra of titanate samples prepared at different calcination temperatures. The spectra reveal an intriguing phenomenon: as the calcination temperature increases, the absorption of visible light by the samples first decreases, then increases. Notably, the sample prepared at 600 °C exhibits the highest absorption of visible light due to the presence of some anatase phase TiO_2_ in the sample, thereby enhancing the absorption of visible light. Interestingly, the nanobelt sample prepared at 650 °C shows slightly stronger absorption of visible light than the sample at 700 °C. This could be attributed to the larger specific surface area of the 650 °C sample, which revealed a redshift in the absorption edge, indicating improved light absorption capability.

The catalyst’s band gap energies are calculated using the Kubelka–Munk method:

$${\left(\alpha h\nu \right)}^{1/n}=A\left(h\nu -E_{g}\right)$$

where α, *h*, *v,* and Eg represent the absorption coefficient, Planck’s constant, frequency, and band gap energy, respectively. A is a constant, and n is considered 1/2 for the semiconductor with a direct gap [46]. Figure 6b shows that we estimated the bandgap widths of the titanium salt samples prepared at different calcination temperatures based on the graph of absorbed light energy against (Ahv)1/2. Table 2 shows these bandgap widths: 2.91, 3.20, 3.25, 3.15, and 3.09 eV, respectively. We observed that the bandgap width shows a pattern of increasing and decreasing. Moreover, from the perspective of the band gap, the bandwidth of 650 °C to 800 °C makes it difficult to support visible light absorption. We speculate that a crossed band is formed between different titanates. Due to the difficulty of separating the composite titanates, the measured band gap is between the highest CB and the lowest VB, rather than the band gap energy that is truly excited by visible light. In addition, there is also some deviation between theory and practice, thus influencing each other and leading to this trend.

**Figure 7:**
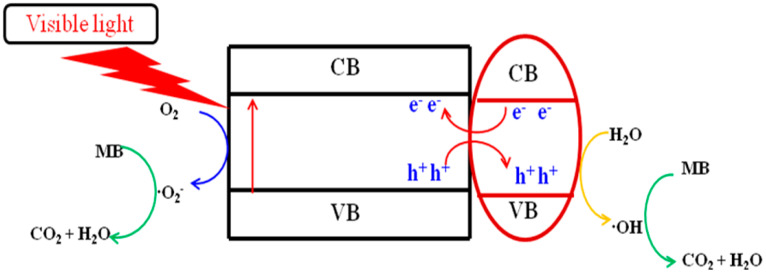
The mechanism for photocatalytic degradation of MB in a titanate sample calcined at 650 °C under visible light irradiation.

Based on the experimental analysis results above and previous work [47,48,49], a possible photocatalytic reaction mechanism for the degradation of MB under visible light by titanium salts prepared at 650 °C calcination temperature is projected, as shown in Figure 7. Under visible light, the electrons of titanium salts transition from the valence band to the conduction band, and the difference in the position of the valence band and conduction band of the two titanium salts causes the migration of electrons and holes between them. The holes left on the valence band migrate to a lower position on the valence band, and the electrons on the conduction band migrate to a higher position on the conduction band, effectively separating photogenerated carriers. The electrons in the CB capture the dissolved oxygen reaction in the solution to form the •O^2−^ radical. Photogenerated holes directly degrade MB adsorbed on the catalyst’s surface or combine with adsorbed water to generate •OH free radicals. Finally, they participate in the photodegradation process of MB.

## 4. Conclusions

This study presents a successful, innovative approach to synthesizing a composite titanate photocatalyst through molten salt processing at different calcination temperatures. The resulting composite material exhibits superior photocatalytic properties compared with single-phase titanate materials. The enhanced photocatalytic activity is attributed to the increased surface area and improved light absorption, which promotes efficient charge separation and pollutant degradation. The synthesis of the composite titanate photocatalyst through molten salt processing offers a promising approach to enhance photocatalytic properties, making it a valuable candidate for a wide range of applications in pollution control, water treatment, and solar energy conversion.

The composite photocatalyst exhibited enhanced photocatalytic properties in MB degradation under visible light and obtained the following conclusions: (1) titanium salt nanobelt samples were successfully prepared at 650 °C and 700 °C via the salt melting method; (2) as the calcination temperature increased, the specific surface area of the titanium salt samples decreased, indicating that temperature can be used to control the specific surface area of our prepared samples; (3) as the calcination temperature increased, the forbidden band width of the titanium salt samples increased first and then decreased; the sample prepared at 600 °C had the smallest forbidden bandwidth, while that prepared at 700 °C had the largest; and (4) the titanium salt sample prepared at 650 °C had a layered structure and a composite structure of nanobelts and nanoparticles. The photocatalytic efficiency of the titanium salt sample prepared at 650 °C was shown to reach 95% MB degradation. In summary, this work provides some ideas for the application of titanium salts in the field of photocatalysis and opens new avenues for the development of advanced photocatalytic materials with improved performance.

## Figures and Tables

**Table 1:** Structural properties of titanate samples calcined at different temperatures.

Temperature	S_BET_ ^a^ (m^2^·g^−1^)	PV ^b^ (cm^3^)	PD ^c^ (nm)
600 °C	76.98	0.268	13.92
650 °C	23.95	0.066	18.78
700 °C	7.86	0.045	22.94
750 °C	2.17	0.013	23.20
800 °C	2.01	0.012	24.67

a: Specific surface area; b: pore volume; c: pore diameter.

**Table 2:** Band gap energy of titanate samples calcined at different temperatures.

Temperature	600 °C	650 °C	700 °C	750 °C	800 °C
E_g_ (eV)	2.91	3.20	3.25	3.15	3.09

## Data Availability

Research data can be provided upon request.
